# Perinatal depression at the intersection of race/ethnicity and disability

**DOI:** 10.1007/s00737-025-01593-y

**Published:** 2025-06-20

**Authors:** Brandie Bentley, Willi Horner-Johnson, Nichole Nidey, Tuyet-Mai Hoang, Chi-Fang Wu, Skky Martin, Ashley Brevil, Reshawna Chapple, Karen M. Tabb

**Affiliations:** 1 Department of Physical Medicine and Rehabilitation, University of Michigan, 325 E Eisenhower Pkwy, Ann Arbor, MI 48108 United States; 2 Institute On Development and Disability, Oregon Health & Science University, Portland, Oregon, United States; OHSU-PSU School of Public Health, Portland, OR United States; 3 College of Public Health, Department of Epidemiology, The University of Iowa, Iowa City, IA United States; 4 School of Social Work, University of Illinois at Urbana-Champaign, Champaign, IL United States; 5 NORC at the University of Chicago, Chicago, IL United States; 6 School of Public Administration, University of Central Florida, Orlando, FL United States; 7 School of Social Work, University of Central Florida, Orlando, FL United States

**Keywords:** Perinatal depression, Race/Ethnicity, Disability, Intersectionality, PRAMS

## Abstract

**Purpose:**

Perinatal depression disparately impacts diverse groups, with marginalized populations often facing greater vulnerability. While previous research has highlighted disparities in perinatal depression by race/ethnicity and by disability status independently, there is a lack of research examining the intersectionality of these social identities and their combined association with perinatal depression. Therefore, this study adopts an intersectional lens to explore variations in perinatal depressive symptoms associated with the combination of race/ethnicity and disability status in a nationally representative sample of women who had recently given birth.

**Methods:**

We conducted a cross-sectional secondary data analysis using 2019–2020 data from the Pregnancy Risk Assessment Monitoring System (PRAMS), including a sample of disabled and non-disabled individuals across 22 sites. We used logistic regression analyses to estimate associations of race/ethnicity and disability status with perinatal depressive symptoms, performing separate analyses for the antenatal and postpartum periods. In adjusted regression models, we controlled for other sociodemographic characteristics.

**Results:**

Our analysis included 33,854 individuals, including 31,480 (93%) without a disability and 2,374 (7%) with at least one disability. Women with disabilities reported higher prevalence of antenatal (42.7%) and postpartum (33.1%) depressive symptoms compared to non-disabled women (14.1% and 12.1%, respectively). Antenatal depression was most common among disabled Non-Hispanic (NH) White women, while prevalence and odds of postpartum depression were highest among disabled NH American/Indian Alaska Native and disabled NH Black women.

**Conclusion:**

Our findings emphasize the need for perinatal depression screening for disabled women, as well as culturally appropriate interventions to support the mental health of diverse women with disabilities throughout the perinatal period.

## Article highlights

• Women with disabilities, across all racial/ethnic groups, reported significantly higher prevalence and odds of both antenatal and postpartum depressive symptoms compared to non-disabled NH White women.

• Postpartum depressive symptoms were markedly more common among NH American Indian/Alaska Native women with disabilities and NH Black women with disabilities than for women with just one of those identities, suggesting compounded disparity associated with the combination of race and disability.

• This study serves as an important step in advancing intersectional maternal mental health research and calls for future studies to further investigate the contextual and structural factors that contribute to the mental health disparities experienced by disabled women of color during the perinatal period.

## Introduction

Perinatal depression is a major public health concern that has serious implications for those who experience it, their children and families, and the broader society. It encompasses depressive symptoms that occur during pregnancy, known as antenatal depression, or within the first 12 months after giving birth, referred to as postpartum depression (ACOG 2018). Symptoms of perinatal depression can include persistent feelings of sadness, hopelessness, loss of interest or pleasure in activities, difficulty bonding with the baby, doubts about caregiving abilities, and thoughts of harming self or baby (NIMH n.d.).

Studies have shown that perinatal depression rates vary significantly across different groups, with individuals from marginalized backgrounds experiencing higher risk of antenatal and postpartum depression (Bauman et al. 2020; Gavin et al. 2011; Liu and Tronick 2013; Mitra et al. 2015). For example, women of color, including American Indian, Alaska Native, Asian, Black/African American, and Hispanic women, have been found to experience higher rates of antenatal and postpartum depression compared to their White counterparts, with differences partially explained by sociodemographic factors such as age, income, and education level (Bauman et al. 2020; Gavin et al. 2011; Liu & Tronick 2013). Similarly, people with disabilities* (Add footnote: Terminology regarding disability is evolving, and both person-first and identity-first phrasings are currently considered acceptable. Since many disabled people prefer identity-first language while others prefer person-first language, we have intentionally used a mix of both throughout the paper (Andrews et al. (2022). The evolution of disability language: Choosing terms to describe disability. *Disability and Health Journal*, 15(3)101328. 10.1016/j.dhjo.2022.101328))* are at a greater risk of experiencing perinatal depressive symptoms, which may be associated with stigma, fragmented social support networks, increased stress, lack of healthcare access, and other unmet needs for care during the perinatal period (Alhusen et al. 2023; Booth et al. 2021; Mitra et al. 2015).

Race and disability are complex social constructs that have historically been used to define, segregate, and oppress (Gillborn 2015). Research shows racism and ableism can have a significant impact on perinatal depression and maternal health. Although women of color experience higher rates of perinatal depressive symptoms and hospitalization, they are less likely to be screened and diagnosed (Chan et al. 2021; Gavin et al. 2011; Sidebottom et al. 2021). Similarly, healthcare providers may have biases or lack knowledge about needs and strategies for perinatal depression screening and care for disabled women, which can lead to disparities in diagnosis and access to adequate mental health care and support (Matin et al. 2021). Because it is less likely to be identified and treated in a timely way, perinatal depression can have particularly devastating impacts for women of color and women with disabilities.

Despite available research on disparities in perinatal depression, there is limited literature that examines the extent to which the prevalence of symptoms varies by race/ethnicity among disabled women. Existing research does not consider how different social identities, such as race/ethnicity and disability status, intersect with each other and with other factors to influence women’s perinatal mental health experiences and outcomes. It is important to study risk factors that contribute to elevated depressive symptoms among diverse groups to effectively reduce disparities in maternal mental health.

In this study, we utilized an intersectional framework combined with Disability Critical Race Theory (DisCrit), to explore how prevalence and odds of perinatal depressive symptoms differ by race/ethnicity and disability status. The intersectionality framework was established as a conceptual and analytical tool that seeks to understand the intersection of race, gender, and other marginalized identities, including disability (Crenshaw 2013). DisCrit encourages a macro-level analysis of how systemic racism and ableism intersect to create a system of oppression that creates additional barriers and contexts that must be considered for dually minoritized populations (Annamma et al. 2013). We hypothesized that, because they face a combination of both racism and ableism, disabled racial/ethnic minority women would have particularly high odds of antenatal depressive symptoms and postpartum depressive symptoms – greater than those for either non-disabled women in the same racial/ethnic group or disabled White women.

## Methods

### Data source and study population

We conducted a cross-sectional secondary data analysis utilizing data from the Pregnancy Risk Assessment Monitoring System (PRAMS, survey years 2019 and 2020). PRAMS is a surveillance project conducted in partnership between the Centers for Disease Control and Prevention (CDC) and state health departments with the goal of decreasing maternal and infant morbidity and mortality (Shulman et al. 2018). PRAMS captures population-based data on maternal attitudes, experiences, and healthcare utilization before, during, and shortly after pregnancy. The questionnaires are linked to birth certificate data that provides additional demographic and medical information. PRAMS oversamples sub-populations by characteristics of public health interest such as infant birth weight, maternal race and ethnicity, Medicaid status, and geography. This process ensures that there is adequate data available for smaller, high-risk populations. States must achieve a response rate > 50% to be included in the dataset released by CDC. Further, PRAMS utilizes a weighting process to ensure that results are representative of the broader U.S. population of individuals who have recently given birth. The PRAMS sample includes individuals assumed to have been assigned “female” at birth; however, we use a mix of the terms “women” and “individuals” to encompass the diverse spectrum of birthing genders and experiences. The study population included respondents from 22 PRAMS sites that met the required CDC response threshold (≥ 50%), collected detailed data on race and ethnicity, and chose to include the supplemental disability questionnaire in either 2019 or 2020 (D’Angelo et al. 2020). Because our study used existing, deidentified data, the Institutional Review Board of the [redacted for blind review] determined that this study was non-human subjects research.

## Measures and analyses

### Dependent variables

The key dependent variable, perinatal depression, includes participant reports of either antenatal depressive symptoms or postpartum depressive symptoms. To assess antenatal depressive symptoms, participants were asked, *“During your most recent pregnancy, did you have any of the following health conditions? …Depression.''* Response options for this question were binary (Yes/No). This measure is not confirmed with a medical diagnosis.

For postpartum depressive symptoms, participants were assessed using a modified Patient Health Questionnaire-2 (PHQ-2) that includes two questions: 1) *“Since your new baby was born, how often have you felt down, depressed, or hopeless?”* and 2) *“Since your new baby was born, how often have you had little interest or pleasure in doing things you usually enjoyed?”* Response options for both questions included always, often, sometimes, rarely, and never. PRAMS created an indicator variable for PPD symptoms (Yes/No) determined by mothers responding always or often to either question (Yes) versus sometimes, rarely, or never to both questions (No)**.**

### Independent variable

**Fig. 1:**
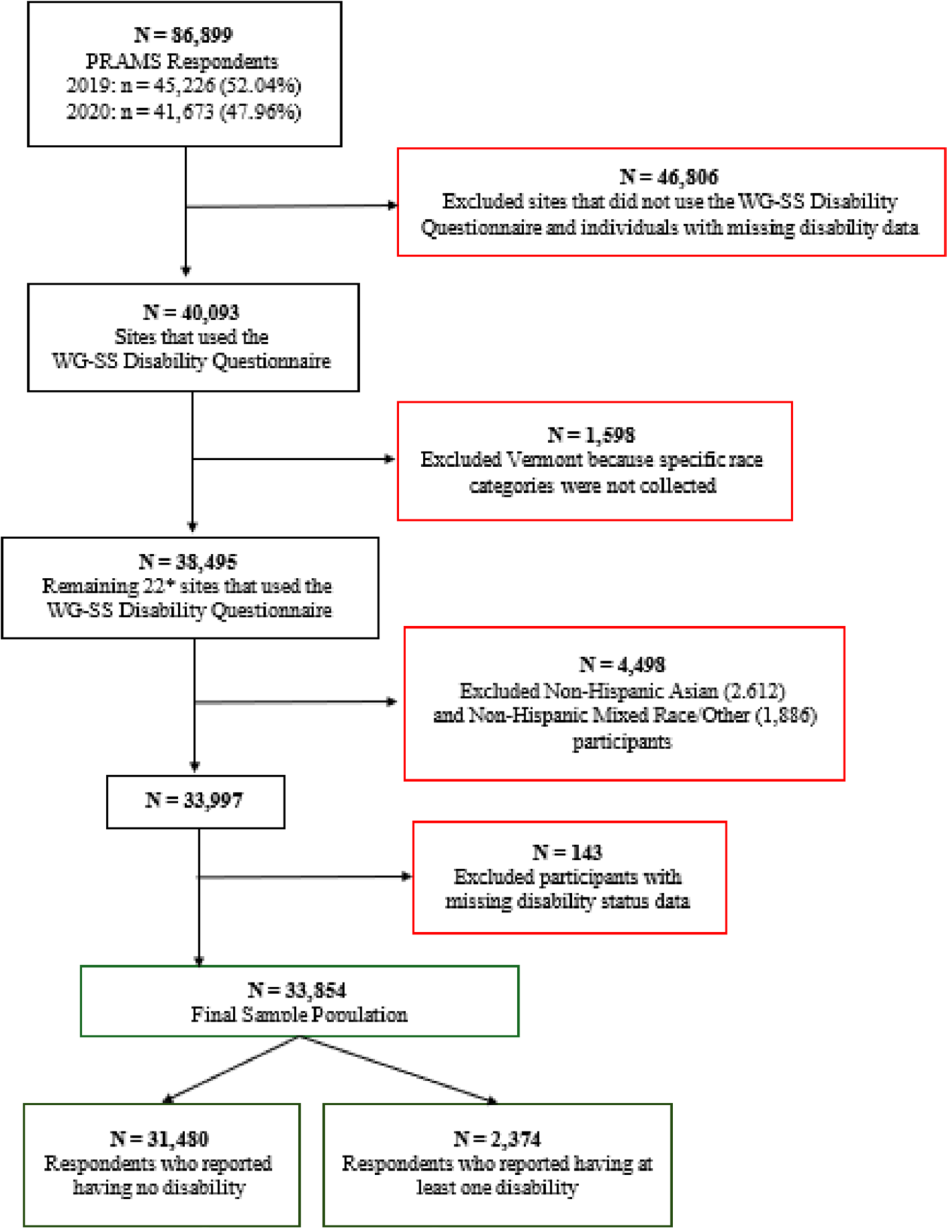
Unweighted Study Sample from 2019 & 2020 PRAMS. *22 Sites Included: Colorado (2019, 2020), District of Columbia (2019, 2020), Georgia (2019, 2020), Kansas (2019, 2020), Louisiana (2019, 2020), Massachusetts (2019, 2020), Maryland (2019, 2020), Maine (2019), Michigan (2019, 2020), Missouri (2019, 2020), Mississippi (2019, 2020), Montana (2019, 2020), North Dakota (2019, 2020), Nebraska (2019, 2020), New Hampshire (2020), New Mexico (2019, 2020), New York City (2019), Oregon (2019, 2020), Rhode Island (2019), South Dakota (2019, 2020), Tennessee (2020), Virginia (2019, 2020)

For this study, we created a combined race/ethnicity and disability status variable. Maternal race and ethnicity were obtained from birth certificates linked to the PRAMS dataset. Maternal race categories available in the PRAMS data included: Alaska Native, American Indian, Black, Chinese, Filipino, Hawaiian, Japanese, Mixed Race, Other Asian, and Other Non-White. Respondents also reported whether they identified as Hispanic ethnicity. To ensure sufficient cell sizes for analysis, we focused on racial/ethnic categories with at least 200 disabled respondents, excluding NH Asian (Chinese, Filipino, Hawaiian, Japanese, and Other Asian) respondents. Additionally, we excluded the Mixed Race and Other categories due to their heterogeneity making it difficult to accurately define who is included within these groups. As a result, these groups were not included in the final analyses (Fig. 1).

PRAMS uses a supplemental disability questionnaire consisted of the Washington Group Short Set of Questions on Disability (WG-Short Set) to assess participant disability status. The WG-Short Set is a cognitively tested validated instrument. It has six questions regarding functional ability that assess difficulty with vision, hearing, mobility, remembering or concentration, self-care, and communicating. The response options measure the severity of an individual’s reported disability status (Madans et al. 2011). Response options for each question include: No difficulty (1), Some difficulty (2), A lot of difficulty (3), or I cannot do this at all (4). For this study, a dichotomous disability variable (Yes/No) was created by combining responses to each WG-Short Set question. For purposes of reporting and generating internationally comparable data, the Washington Group recommends using the “Disability 3” cutoff for defining the population of people with disabilities (Washington Group on Disability Statistics n.d.). Therefore, a response of A lot of difficulty (3) or I cannot do this at all (4) on any of the six questions was recoded as “Disabled”, while No difficulty (1) and Some difficulty (2) were recoded as “Not Disabled.”

To examine the intersection of disability and race/ethnicity, we created a combined variable with the following eight categories: NH American Indian/Alaska Native (AI/AN) Disabled (1), NH AI/AN Non-Disabled (2), NH Black Disabled (3), NH Black Non-Disabled (4), Hispanic Disabled (5), Hispanic Non-Disabled (6), NH White Disabled (7), and NH White Non-Disabled (referent category) (8).

### Covariates

Our analyses considered several additional sociodemographic variables that previous research has found to be associated with an increased risk of experiencing perinatal depression. These are maternal age (< = 19, 20–29, 30–39, 40 +), education level (less than high school, high school or equivalent, some college, college degree or higher), insurance type (public, private, other, uninsured), and marital status (married, other) (Alhusen et al. 2023; Booth et al. 2021; Mitra et al. 2015).

### Statistical analysis

Data analysis was performed using SAS 9.4 software. For all analyses, we applied PRAMS analysis weights that account for sampling, nonresponse, and noncoverage (Shulman et al. 2018). Due to the PRAMS quality control measures, item nonresponse rates are typically low, ranging from 1–2% for most questions. Therefore, no imputation procedures are used for item nonresponse (Shulman et al. 2018). We conducted complete case analyses such that all respondents with non-missing data for the variables in question were included (e.g., respondents were included in the antenatal depression analysis if they had data on antenatal depression, regardless of whether or not they also had data on postpartum depression). P-values ≤. 0.05 were considered statistically significant.

Descriptive statistics were conducted to examine study population demographic characteristics. We conducted bivariate and multivariable logistic regression models to test the associations between our combined race/ethnicity by disability status variable and perinatal depression, using separate models for the antenatal and postpartum periods. Our multivariable models adjusted for sociodemographic characteristics on which our groups differed significantly and that have previously been found to be associated with perinatal depression.

## Results

Our analytic sample included 33,854 individuals meeting our inclusion criteria (Fig. 1), of whom 31,480 (93%) were non-disabled, and 2,374 (7%) reported having at least one disability. NH White women (52.9%) had the largest sample size, followed by NH Black (21.8%), Hispanic (19.8%), and NH AI/AN (5.5%).

Table 1 presents the demographic characteristics, stratified by race/ethnicity and disability status. Women with disabilities tended to be younger than their non-disabled counterparts, with more than half being 29 years old or younger. Cognitive disability was the most prevalent disability type, ranging from 48.6% to 66.2% across all racial/ethnic groups. Disabled women in all racial/ethnic groups were less likely to have completed college or higher education compared to non-disabled women. Notably, non-disabled NH White women had the highest proportion that had completed college (49.2%), while disabled NH AI/AN women had the lowest (1.0%). Individuals with disabilities in all groups were more likely to have public insurance compared to those without disabilities. Lastly, NH White non-disabled women were the most likely to be married (74.4%). The percentages may not sum to 100% due to rounding. Percentages for disability type add up to more than 100% because respondents could indicate more than one type of disability Missingness rates are as follows: Age (n = 5, 0.01%), Education Level (n = 201, 0.59%), Insurance Type (n = 1,830, 5.41%), and Marital Status (n = 15, 0.04%) P-values calculated using Chi-Squared tests

**Table 1:** Demographic Characteristics of Sample Population, Stratified by Race/Ethnicity and Disability Status, 2019–2020 PRAMS (N = 33,854) (Unweighted n, Weighted %)

	NH AI/AN (n = 1,877)		NH Black (n = 7,373)		NH White (n = 17,914)		Hispanic (n = 6,690)	
	Disability Status		Disability Status		Disability Status		Disability Status	
Variable	No (n=1,654)	Yes (n=223)	No (n=6,799)	Yes (n=574)	No (n=16,838)	Yes (n=1,076)	No (n=6,189	Yes (n=501)
	n (%)	n (%)	n (%)	n (%)	n (%)	n (%)	n (%)	n (%)
Age (years)								
< = 19	164 (9.9)	19 (6.8)	404 (5.7)	48 (7.6)	390 (3.0)	56 (4.9)	399 (6.4)	39 (7.3)
20–29	936 (52.7)	125 (49.0)	3,441 (53.4)	296 (52.3)	6,996 (44.0)	566 (55.2)	3,219 (54.3)	283 (62.4)
30–39	521 (35.6)	75 (28.2)	2,663 (37.3)	217 (36.8)	8,914 (50.4)	419 (37.2)	2,337 (35.7)	157 (24.8)
40+	33 (1.8)	4 (16.0)	290 (3.5)	13 (3.2)	536 (2.8)	35 (2.7)	232 (3.5)	22 (5.5)
Disability Type								
Vision	--	93 (42.7)	--	219 (38.7)	--	245 (22.4)	--	161 (29.9)
Hearing	--	23 (8.6)	--	63 (10.9)	--	102 (9.4)	--	41 (10.7)
Mobility	--	20 (10.1)	--	99 (16.5)	--	102 (8.2)	--	47 (9.7)
Cognitive	--	109 (52.9)	--	274 (48.6)	--	710 (66.2)	--	249 (52.6)
Self-Care	--	10 (4.8)	--	37 (4.3)	--	41 (3.8)	--	31 (9.6)
Communication	--	26 (9.4)	--	74 (11.2)	--	65 (5.0)	--	83 (20.1)
Educational level								
< High School	422 (25.4)	63 (24.3)	785 (10.8)	93 (15.2)	818 (6.3)	140 (13.7)	1,669 (30.0)	147 (34.5)
High School or Equivalent	511 (32.7)	72 (44.1)	2,288 (36.1)	221 (43.0)	2,782 (20.1)	329 (33.0)	1,771 (32.0)	162 (34.5)
Some College	573 (32.8)	82 (30.7)	2,313 (32.0)	197 (33.7)	4,383 (24.5)	382 (33.5)	1,618 (22.9)	142 (21.4)
≥ College Degree	140 (9.2)	5 (1.0)	1,375 (21.1)	54 (8.1)	8,803 (49.2)	219 (19.9)	1,051 (15.1)	43 (9.6)
Insurance Type								
Public	854 (62.1)	142 (65.7)	2,761 (40.31)	309 (46.8)	2,113 (15.2)	355 (36.8)	1,630 (25.1)	199 (37.0)
Private	329 (28.1)	31 (27.6) 23 (8.6)	2,545(39.7)	146 (35.8)	12,144 (72.7)	464 (43.0)	2,111 (30.6)	120 (28.2)
Other	27 (1.2)	4 (0.8)	252 (4.1)	26 (4.5)	511 (3.1)	50 (4.8)	251 (4.5)	20 (5.0)
Uninsured	161 (8.6)	16 (6.0)	873 (16.0)	74 (13.0)	1,383 (9.0)	160 (15.4)	1,836 (39.8)	127 (29.8)
Marital Status								
Married	359 (23.9)	44 (31.1)	2,127 (30.2)	142 (29.0)	12,974 (74.4)	516 (47.3)	3,091 (47.3)	199 (39.5)
Other	1,295 (76.1)	179 (68.9)	4,668 (69.80)	432 (71.0)	3,857 (25.6)	559 (52.7)	3.096 (52.7)	301 (60.5)

NH Non-Hispanic

AI/AN American Indian/Alaska Native

The percentages may not sum to 100% due to rounding. Percentages for disability type add up to more than 100% because respondents could indicate more than one type of disability

Missingness rates are as follows: Age (n = 5, 0.01%), Education Level (n = 201, 0.59%), Insurance Type (n = 1,830, 5.41%), and Marital Status (n = 15, 0.04%)

**Fig. 2:**
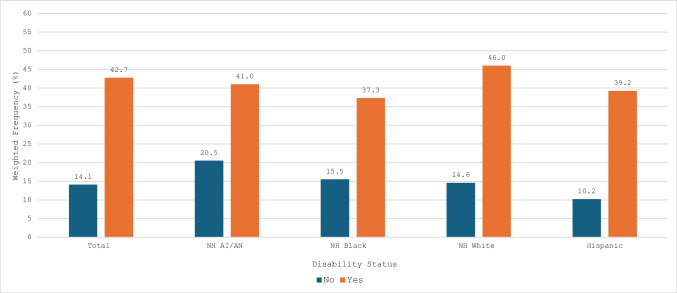
Prevalence of Antenatal Depressive Symptoms Stratified by Race/Ethnicity and Disability Status, 2019–2020 PRAMS (N = 33,431)

Figure 2 shows the prevalence of antenatal depressive symptoms, stratified by race/ethnicity and disability status. Overall, 14.1% of non-disabled individuals reported experiencing antenatal depressive symptoms. The prevalence was significantly higher among women with disabilities, at 42.7%. Nearly half of NH White women with disabilities (46%) reported experiencing antenatal depressive symptoms. In contrast, non-disabled Hispanic women had the lowest prevalence, with only 10.2% reporting symptoms.

In our unadjusted regression model examining the associations of race/ethnicity and disability status with antenatal depressive symptoms, women with disabilities across all racial/ethnic groups had significantly higher odds of experiencing antenatal depressive symptoms compared to non-disabled NH White women (Table 2, Model 1). The magnitude of the odds ratio was largest for NH White women with disabilities (OR = 4.99; 95% CI: 4.12, 6.04). After adjusting for maternal age, education level, insurance type, and marital status (Table 4, Model 2), the odds remained elevated but were attenuated. NH AI/AN women with disabilities had an adjusted odds ratio (aOR) of 2.23 (95% CI: 1.29, 3.87), NH Black women with disabilities had an aOR of 2.01 (95% CI: 1.43, 2.80), and Hispanic women with disabilities had an aOR of 2.53 (95% CI: 1.67, 3.85). In contrast, non-disabled NH Black women (aOR = 0.66; 95% CI: 0.56, 0.77) and non-disabled Hispanic women (aOR = 0.49; 95% CI: 0.41, 0.58) had significantly lower odds of antenatal depressive symptoms compared to non-disabled NH White women.

**Table 2:** Unadjusted and Adjusted Logistic Regression Models Examining Associations of Race/Ethnicity & Disability Status with Antenatal Depressive Symptoms, 2019–2020 PRAMS

	Model 1: Unadjusted (n = 33,431)	Model 2: Adjusted (n = 31,450)
Maternal Race/Ethnicity & Disability Status	OR (95% CI)	aOR (95% CI)
NH AI/AN Disabled	**4.07 (2.18–7.60)**	**2.23 (1.29–3.87)**
NH AI/AN Non-Disabled	**1.51 (1.13–2.01)**	0.81 (0.58–1.12)
NH Black Disabled	**3.48 (2.58–4.69)**	**2.01 (1.43–2.80)**
NH Black Non-Disabled	1.07 (0.94–1.23)	**0.66 (0.56–0.77)**
Hispanic Disabled	**3.78 (2.57–5.53)**	**2.53 (1.67–3.85)**
Hispanic Non-Disabled	**0.67 (0.57–0.78)**	**0.49 (0.41–0.58)**
NH White Disabled	**4.99 (4.12–6.04)**	**3.71 (3.04–4.53)**
NH White Non-Disabled	**REF**	**REF**

*NH* Non-Hispanic

*AI/AN* American Indian/Alaska Native

*CI* Confidence Interval

Bold values are statistically significant

Model 1: Unadjusted model examining the associations of race/ethnicity and disability status with antenatal depressive symptoms

Model 2: Adjusted for maternal age, education level, insurance type, and marital status

**Fig. 3:**
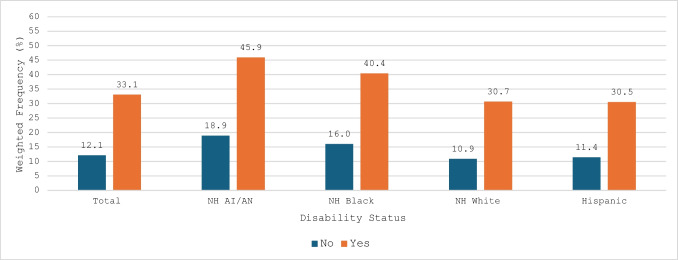
Prevalence of Postpartum Depressive Symptoms Stratified by Race/Ethnicity and Disability Status, 2019–2020 PRAMS (N = 33,520)

The overall prevalence of postpartum depressive symptoms was slightly lower than antenatal depressive symptoms, for both non-disabled (12.1%) and disabled (33.1%) participants (Fig. 3). Prevalence was higher among women with disabilities, ranging from 30.5% to 45.9% across racial/ethnic groups. NH AI/AN women with disabilities had the highest prevalence (45.9%), while non-disabled NH White women reported the lowest prevalence (10.9%).

For postpartum depressive symptoms, both unadjusted and adjusted models showed significant disparities by race/ethnicity and disability status. In the unadjusted model (Table 3, Model 1), women with disabilities in all racial/ethnic minority groups had significantly higher odds of postpartum depressive symptoms compared to non-disabled NH White women. The magnitude of the odds ratio was largest for NH AI/AN women with disabilities (OR = 6.92; 95% CI: 3.32, 14.40), followed by NH Black women with disabilities (OR = 5.54; 95% CI: 4.09, 7.50). After adjusting for sociodemographic variables, disabled women still showed higher odds of postpartum depression, though the associations were attenuated. Notably, among women without disabilities, Hispanic women had significantly lower odds of postpartum depressive symptoms compared to NH White women without disabilities (aOR = 0.82; 95% CI: 0.68, 0.98). In contrast, the highest adjusted odds were observed among NH AI/AN women with disabilities (aOR = 5.19; 95% CI: 2.04, 13.20), followed by NH Black women with disabilities (aOR = 3.78; 95% CI: 2.73, 5.22) (Table 3, Model 2). Importantly, in the disabled NH AI/AN and NH Black groups, the size of the aORs were larger than those for their non-disabled counterparts of the same race/ethnicity or for NH White disabled women, when comparted to NH White non-disabled women.

**Table 3:** Unadjusted and Adjusted Logistic Regression Models Examining Associations of Race/Ethnicity & Disability Status with Postpartum Depressive Symptoms, 2019–2020 PRAMS

	Model 1: Unadjusted (n = 33,520)	Model 2: Adjusted (n = 31,521)
Maternal Race/Ethnicity & Disability Status	OR (95% CI)	aOR (95% CI)
NH AI/AN Disabled	**6.92 (3.32–14.40)**	**5.19 (2.04–13.20)**
NH AI/AN Non-Disabled	**1.90 (1.42—2.53)**	1.24 (0.90–1.17)
NH Black Disabled	**5.54 (4.09–7.50)**	**3.78 (2.73–5.22)**
NH Black Non-Disabled	**1.55 (1.40–1.77)**	1.09 (0.93–1.27)
Hispanic Disabled	**3.58 (2.35–5.46)**	**1.96 (1.33–2.90)**
Hispanic Non-Disabled	1.05 (0.89–1.24)	**0.82 (0.68–0.98)**
NH White Disabled	**3.62 (2.95–4.44)**	**2.74 (2.20–3.42)**
NH White Non-Disabled	**REF**	**REF**

*NH* Non-Hispanic

*AI/AN* American Indian/Alaska Native

*CI* Confidence Interval

Bold values are statistically significant

Model 1: Unadjusted model examining the associations of race/ethnicity and disability status with postpartum depressive symptoms

Model 2: Adjusted for maternal age, education level, insurance type, and marital status

## Discussion

This is one of the first studies to our knowledge that examines the association of both race/ethnicity and disability with perinatal depression, using nationally representative data. Our findings advance the application of DisCrit Theory in perinatal mental health research and provide an essential foundation for intersectional frameworks to be applied more in maternal health scholarship.

**Fig. 4:**
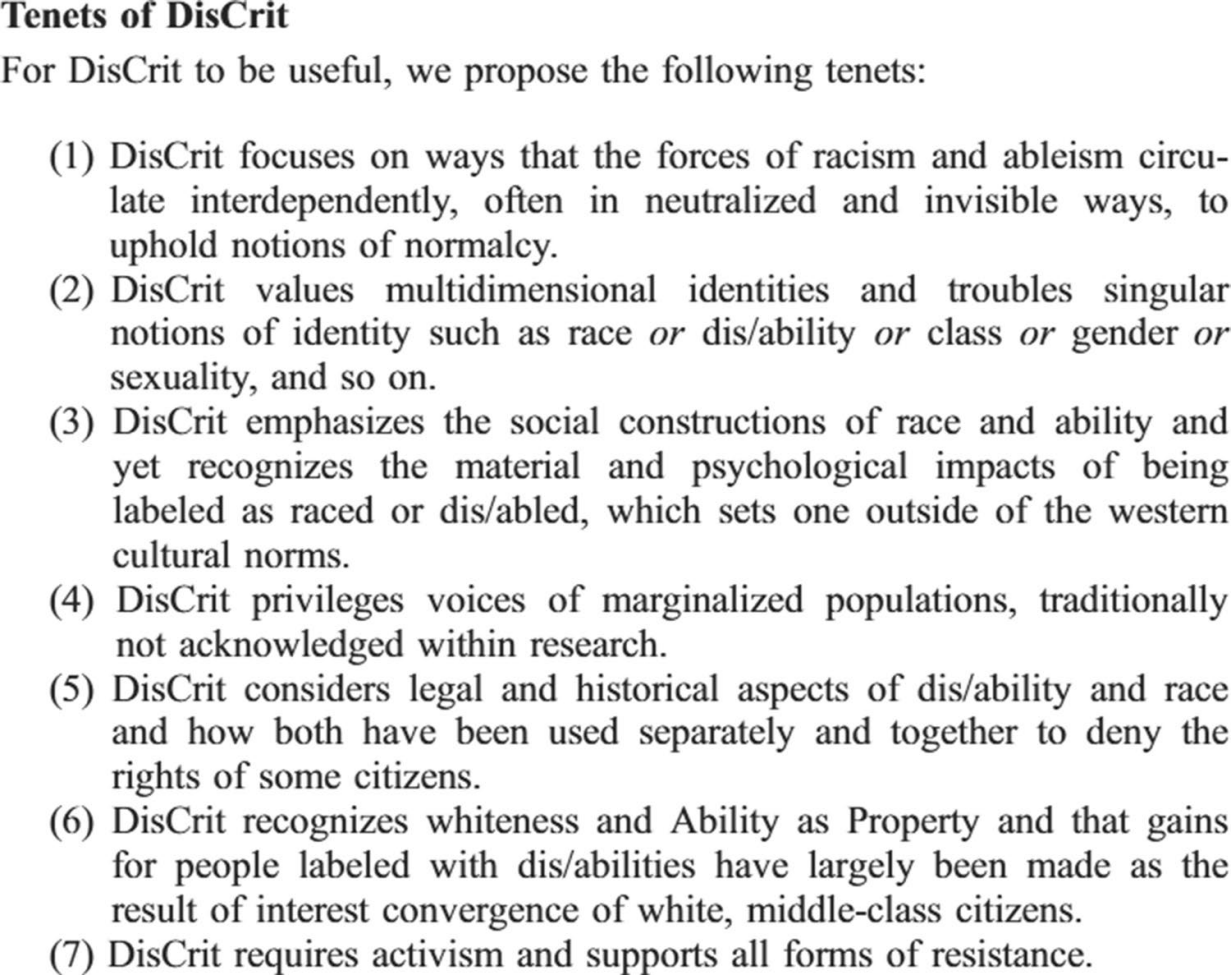
Key Tenets of DisCrit

The key tenets of DisCrit are shown in Fig. 4. Specifically, tenets two, four, and seven informed this study’s conceptualization and interpretation. Tenet two emphasizes that identities such as race, ethnicity, and disability are interdependent and must be examined in relation to one another. Overall, we found that a significant percentage of disabled women experienced antenatal (42.7%) and postpartum depressive symptoms (33.1%). These findings are consistent with previous research, which has shown that women with disabilities in the United States face a higher risk of perinatal depression compared to those without disabilities (Alhusen et al. 2023; Booth et al. 2021; Mitra et al. 2015). We expanded on prior research by revealing that this pattern was consistent across all racial/ethnic groups in our study. Results from this study also partially support our hypothesis that disabled racial/ethnic minority women would experience higher odds of depressive symptoms than non-disabled women in the same racial/ethnic group and disabled White women. In the antenatal period, depressive symptoms were actually most common among White women with disabilities, with no evidence of cumulative disparity associated with the combination of race/ethnicity and disability. However, in our postpartum analyses, prevalence and odds of depressive symptoms were highest among NH AI/AN and Black women with disabilities. Importantly, the magnitude of the OR of postpartum depressive symptoms among disabled NH AI/AN women was notably larger than the magnitude of the ORs for NH White disabled women and NH AI/AN non-disabled woman, indicating that the combination of race and disability was associated with markedly greater disparity than either AI/AN race alone or disability status alone. A similar but less pronounced pattern was observed for disabled NH Black women in the postpartum period.

Women with disabilities were consistently more likely to experience perinatal depression than those without disabilities. Unfortunately, many of the depression symptoms women with disabilities experience may remain undiagnosed and untreated. One contributing factor is the lack of disability competency among healthcare providers, which can lead to diagnostic overshadowing, where depressive symptoms are attributed to the person’s disability instead of being recognized as an independent mental health condition. Moreover, widely used screening tools such as the Edinburgh Postnatal Depression Scale and Patient Health Questionnaire may not be accessible to some individuals with disabilities due to differences in language processing, cognition and communication difficulties, or atypical presentations of depression (Brown & Mitra 2024; Shea et al. 2024). These limitations in screening accessibility and accuracy further exacerbate disparities in diagnosis and care. Interestingly, the extent to which there appeared to be additional disparities in depression symptoms associated with race and ethnicity differed for antenatal and postpartum depression. The differences we observed between the antenatal and postpartum periods suggest that the intersection of race, ethnicity, and disability can influence the severity and nature of depressive symptoms in distinct ways during different phases of the perinatal period, highlighting the critical importance of perinatal depression screening at multiple time points for women with disabilities in all racial and ethnic groups.

Tenet four of DisCrit calls for the centering of marginalized voices. By focusing on disabled women of color, a group largely understudied in perinatal mental health research, this study challenges dominant narratives and highlights the urgency of inclusive research, policy, and practice. Future studies with a larger representation of diverse racial/ethnic groups can provide an increased understanding of the prevalence, risk factors, and outcomes associated with perinatal depression for disabled women of color. Findings from this study also promote the importance of continued systematic identification and monitoring of disability status and its association with health outcomes in diverse perinatal populations.

Lastly, tenet seven positions scholarship as a tool for social transformation. Our findings underscore the need for healthcare systems and policies to move beyond documenting disparities and toward implementing intersectional, community-informed, and accessible approaches to care. The disparities revealed in our study highlight the need for targeted interventions and support services that address the unique needs of disabled individuals during the perinatal period. These efforts must focus on identifying and overcoming barriers to accessing comprehensive mental health care and be culturally responsive and inclusive, accounting for the varied experiences and challenges across diverse communities. Existing literature has noted the need for clinicians, researchers, and policymakers to explicitly consider the intersectionality of race/ethnicity and disability when addressing disparities and developing sustainable approaches to promote health equity (Gulley et al. 2014; Horner-Johnson et al. 2021; Mitra et al. 2022; Yee et al. 2018). An intersectional framework is essential in acknowledging and addressing the multiple, overlapping forms of discrimination that shape health outcomes. This study serves as an important step in advancing intersectional maternal mental health research and calls for future studies to further investigate the contextual and structural factors that contribute to the disparities experienced by disabled women of color during the perinatal period.

## Limitations

This study has several limitations that should be considered. First, PRAMS data are self-reported and subject to social desirability and recall bias. Due to a lack of awareness and stigma associated with mental health, PRAMS participants may under report their depressive symptoms. Additionally, the measures used to capture depressive symptoms across different time points within PRAMS do not represent a clinical assessment of depression. Thus, an individual's knowledge of depression, cultural differences, or varied interpretations of the survey questions may result in an inaccurate representation of depressive symptoms in the sample population. Second, sample sizes were too small to stratify our analyses by disability type. People with different types of disabilities may encounter varying socio-environmental barriers that could influence experiences of depression, as well as access to diagnostic and treatment services. Third, depression may vary in relation to additional factors – such as language and immigration or residency status – that we were unable to include in our analyses. We acknowledge that these factors could potentially influence the results, particularly in relation to access to healthcare, cultural differences, and the impact of immigration status on maternal health. Finally, PRAMS data are cross-sectional, so the study cannot determine causal relationships among variables.

## Conclusions

Perinatal depression presents a significant challenge to public health, with substantial consequences for both maternal and child well-being. This study adds to existing literature by exploring the intersectionality of race/ethnicity and disability status in association with perinatal depression. While our study contributes to understanding the complex interplay between race/ethnicity and disability in association with perinatal depressive symptoms, further research is needed to identify the underlying mechanisms driving these disparities and to inform the development of effective interventions aimed at promoting positive mental health and well-being during the perinatal period for all individuals, regardless of race, ethnicity, or disability status.
